# New Aspects of the Pathogenesis of Canine Distemper Leukoencephalitis

**DOI:** 10.3390/v6072571

**Published:** 2014-07-02

**Authors:** Charlotte Lempp, Ingo Spitzbarth, Christina Puff, Armend Cana, Kristel Kegler, Somporn Techangamsuwan, Wolfgang Baumgärtner, Frauke Seehusen

**Affiliations:** 1 Department of Pathology, University of Veterinary Medicine, Bünteweg 17, Hannover D-30559, Germany; E-Mails: charlotte.lempp@tiho-hannover.de (C.L.); ingo.spitzbarth@tiho-hannover.de (I.S.); christina.puff@tiho-hannover.de (C.P.); armend.cana@tiho-hannover.de (A.C.); kristel.kegler@tiho-hannover.de (K.K.); frauke.seehusen@tiho-hannover.de (F.S.); 2 Department of Pathology, Faculty of Veterinary Science, Chulalongkorn University, Bangkok 10330, Thailand; E-Mail: somporn62@yahoo.com

**Keywords:** axon, canine distemper virus, central nervous system, cytokine, distemper leukoencephalitis, immature astrocyte, matrix metalloproteinase, p75 neurotrophin receptor, Schwann cell, vimentin

## Abstract

Canine distemper virus (CDV) is a member of the genus morbillivirus, which is known to cause a variety of disorders in dogs including demyelinating leukoencephalitis (CDV-DL). In recent years, substantial progress in understanding the pathogenetic mechanisms of CDV-DL has been made. *In vivo* and *in vitro* investigations provided new insights into its pathogenesis with special emphasis on axon-myelin-glia interaction, potential endogenous mechanisms of regeneration, and astroglial plasticity. CDV-DL is characterized by lesions with a variable degree of demyelination and mononuclear inflammation accompanied by a dysregulated orchestration of cytokines as well as matrix metalloproteinases and their inhibitors. Despite decades of research, several new aspects of the neuropathogenesis of CDV-DL have been described only recently. Early axonal damage seems to represent an initial and progressive lesion in CDV-DL, which interestingly precedes demyelination. Axonopathy may, thus, function as a potential trigger for subsequent disturbed axon-myelin-glia interactions. In particular, the detection of early axonal damage suggests that demyelination is at least in part a secondary event in CDV-DL, thus challenging the dogma of CDV as a purely primary demyelinating disease. Another unexpected finding refers to the appearance of p75 neurotrophin (NTR)-positive bipolar cells during CDV-DL. As p75NTR is a prototype marker for immature Schwann cells, this finding suggests that Schwann cell remyelination might represent a so far underestimated endogenous mechanism of regeneration, though this hypothesis still remains to be proven. Although it is well known that astrocytes represent the major target of CDV infection in CDV-DL, the detection of infected vimentin-positive astrocytes in chronic lesions indicates a crucial role of this cell population in nervous distemper. While glial fibrillary acidic protein represents the characteristic intermediate filament of mature astrocytes, expression of vimentin is generally restricted to immature or reactive astrocytes. Thus, vimentin-positive astrocytes might constitute an important cell population for CDV persistence and spread, as well as lesion progression. *In vitro* models, such as dissociated glial cell cultures, as well as organotypic brain slice cultures have contributed to a better insight into mechanisms of infection and certain morphological and molecular aspects of CDV-DL. Summarized, recent *in vivo* and *in vitro* studies revealed remarkable new aspects of nervous distemper. These new perceptions substantially improved our understanding of the pathogenesis of CDV-DL and might represent new starting points to develop novel treatment strategies.

## 1. Introduction

Canine distemper virus (CDV) is a single-stranded, non-segmented, negative-sense RNA virus belonging to the genus morbillivirus. It can cause a devastating disease in dogs and other carnivores [1]. In contrast to other paramyxoviruses, such as rinderpest virus, CDV is far beyond eradication [2,3].

In affected dogs, CDV is known to cause a large variety of clinical signs, depending mainly upon the age and immune status of the host as well as the virus strain. Infection can lead to abortive, clinical or subclinical disease courses [3,4]. Similar to other paramyxoviruses, such as the closely related measles virus (MV), CDV infection causes lymphoid depletion and enduring immunosuppression, which favor secondary infections [3]. Clinical signs in affected dogs include catarrhal respiratory and gastrointestinal disorders, alterations of the skin, and central nervous system (CNS) disease [3,5,6,7]. The latter most commonly presents as canine distemper virus induced demyelinating leukoencephalitis (CDV-DL). Morphologically, and especially in terms of immunopathological processes, glial reponses, and evidence of early axonal degeneration, CDV-DL shares certain characteristics with other demyelinating diseases, such as multiple sclerosis (MS) in humans and its experimental animal models [8,9,10]. This is especially true for the chronic phase of CDV-DL, which is characterized by decreased viral replication, paralleled by marked inflammation and demyelination. Several novel and striking features of the pathogenesis of nervous distemper have been detailed only recently. These studies have highlighted alterations in the maturation and plasticity of astrocytes, primary axonopathy, and a potential role of Schwann cell-mediated regeneration as crucial events in CDV-DL. Furthermore, recent observations indicate that a dysregulated orchestration of cytokines, matrix metalloproteinases (MMPs), and their inhibitors might constitute another pivotal factor, influencing the degree and fate of inflammation and virus distribution in CDV-DL. In addition, the establishment of several new *in vitro* models allowed appropriate simulation of certain aspects of CDV-DL pathogenesis.

The aim of this communication is to summarize current trends and recently highlighted aspects in CDV-DL research. Furthermore, consequent future research perspectives with a focus on targets with therapeutic potential in demyelinating diseases will be pointed out.

## 2. Pathogenesis of Canine Distemper

Much of the knowledge on the early phase of CDV infection is based on experimental studies in dogs [11]. More recently, experimental infection of ferrets has been introduced as an appropriate model for studies on CDV infection routes and virus-host cell interactions, respectively [12,13,14,15]. CDV infects dogs mainly oronasally via inhalation of aerosols, whereas transmission via urine and droppings or ingestion of infected meat represent another route of infection, which mainly occurs in wild carnivores [12]. CDV infection of dogs is followed by replication of the virus in lymphoid tissues of the respiratory tract and is predominantly found in local tissue macrophages, which migrate to tonsils and bronchial lymph nodes [4,11]. Subsequently, primary viremia leads to spread into distant lymphoid and hematopoietic tissues, such as spleen, thymus, lymph nodes, and bone marrow, resulting in lymphopenia and immunosuppression, which may provide ground for secondary bacterial infections [3]. Moreover, mucosa-associated lymphatic tissues (MALT) and macrophages in the lamina propria of the gastrointestinal tract may be infected [16]. The subsequent fate of the infection mainly depends on the virulence of the respective CDV strain, the age of the infected individual, and its immune status [4]. A failing or insufficient humoral response during this infection period may promote secondary viremia, while the presence of a robust antiviral immune response may enable the infected individual to eliminate the virus, resulting in recovery [17,18,19]. Secondary viremia can result in virus spread to various epithelial and mesenchymal tissues as well as the CNS [18]. In this stage, CDV mainly infects epithelial targets, such as bronchial and gastrointestinal mucosa, and can additionally be found in keratinocytes, fibroblasts, thrombocytes, different lymphoid cell subsets, and endothelial cells of several parenchymas [3,20]. CNS involvement represents a complication, which often occurs in parallel or subsequently to other organ affections [19].

### Neuropathogenesis of Distemper

Several strains of CDV have a considerable neurotropism. For instance, certain isolates such as the Snyder Hill strain are known to primarily cause acute polioencephalitis, whereas A75/17 and R252 strains predominantly cause demyelinating leukoencephalitis [4,21]. CDV may enter the brain in distinct ways and several routes of infection have been proposed. The main route of neuroinvasion is via infected mononuclear cells trafficking through the blood-brain-barrier (BBB), which results in local virus release and subsequent infection of resident epithelial and endothelial cells [13,22,23]. Moreover, there is also evidence of primary CNS endothelial cell infection contributing to neuroinvasion prior to the trafficking of virus-positive leukocytes [24,25]. In fact, CDV-infected cells are first detected in choroid plexus cells and brain vessels [25,26,27]. Furthermore, there are hints for a spread from cells of the pia mater to the subpial grey matter [3,20,21]. Once inside the brain, the virus spreads via the cerebrospinal fluid (CSF), where it may infect ependymal lining cells of the ventricles and ultimately glial cells and neurons [13,19]. Besides the classical hematogenous route of infection, an additional mechanism of entry has been identified in ferrets, experimentally infected with CDV. Here, the virus has been shown to enter the brain via neurons located in the olfactory mucosa, followed by virus invasion along olfactory nerve filaments to the olfactory glomeruli and subsequent anterograde spread to deeper CNS structures [13]. Whether this anterograde route of infection plays a role in dogs yet remains to be established. Another question that remains to be resolved, is related to the cellular receptors contributing to the neurovirulence of CDV. In fact, signaling lymphocyte activation molecule (SLAM) is expressed to a very limited extent in the CNS [12,28]. Thus, additional and currently unknown receptors may enable the virus to especially invade neural cells [19,28,29].

In dogs, CNS involvement of CDV infection may appear in distinctive manifestations. Grey matter manifestation occurs in rare occasions, and can either present as acute fulminant encephalopathy and encephalitis, respectively, which have for instance been observed in the cerebellar and cerebral cortex in experimentally infected, young and immunologically naïve dogs [3,30,31]. Moreover, post-vaccinal encephalitis (PVE) has been observed following vaccination with a modified live vaccine, affecting the cerebral cortex and brain stem neurons with neuronal necrosis, mononuclear perivascular cuffs, and occasionally intraneuronal inclusion bodies [32,33]. PVE should not be confused with post-vaccination encephalomyelitis in humans, which can for instance occur following vaccination against measles. This rare human disease entity is based on an auto-immune reaction, and in contrast to CDV-related PVE, not associated with virus infection of the brain [34]. A grey matter distribution pattern has additionally been observed in older dogs and was subsequently termed old dog encephalitis (ODE) [35]. This entity however represents an extremely rare complication of CDV infection and due to the paucity of cases the exact pathogenesis and the detailed role of CDV remain elusive [36]. However, ODE is believed to occur in immunocompetent individuals with neuronal persistence of replication-defective virus [37]. In cases of polioencephalitis, which neither have a history of recent vaccination nor affect aged individuals, the rather descriptive term inclusion body polioencephalitis (IBP) has been suggested [38]. However, all forms of polioencephalitis share common features, and thus the exact distinction between the aforementioned types is not always applicable. In contrast to the generally rare polioencephalitic forms, subacute to chronic CDV infection of the CNS can lead to CDV-DL, which represents the most common manifestation of CNS involvement.

## 3. Canine Distemper Leukoencephalitis — Novel Aspects of its Pathogenesis

### 3.1. Pathogenesis and Morphology of Distemper Leukoencephalitis

**Figure 1:**
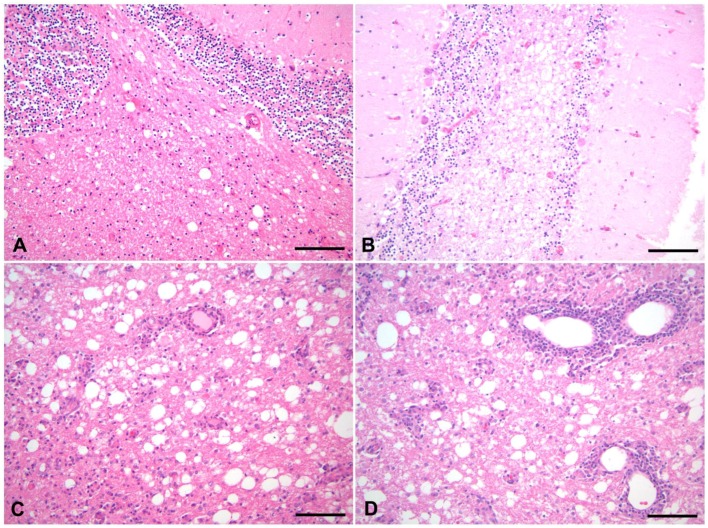
Histological lesions of CDV-DL in the time course of disease. (**A**) Acute lesions are characterized by vacuolization of the white matter due to myelin sheath edema. Inflammation is nearly lacking and restricted to a sparse intraparenchymal infiltration of T cells; (**B**,**C**) Progressive myelin loss (pallor and lack of eosinophilia) is present in subacute lesions. Subacute lesions are further divided into lesions without (**B**) and with (**C**) inflammatory cell infiltration; (**D**) Chronic lesions invariably display more than three layers of perivascular inflammatory cells, accompanying ongoing demyelination and axonal loss. Hematoxylin and eosin staining. Bars = 50 µm.

CDV-DL represents a devastating complication of CDV infection and may occur in parallel with or subsequent to other organ manifestations. It predominantly affects the cerebellum and periventricular regions [3]. CDV-DL commonly starts with infection of the grey matter followed by antigen spread into the white matter [3,25]. Subsequently, CDV-DL follows a certain time course, which strikingly correlates with neuropathological alterations. A well-described histological classification scheme has been used by several authors in order to categorize white matter lesions (Figure 1) [39,40,41]. In this respect, acute, subacute, and chronic lesions are distinguished (Figure 1). These histological stages have been associated with time frames following experimental infection with CDV, which show similar morphological alterations [9]. It has been proposed that acute lesions may correspond to days 16–24, subacute lesions to days 24–32, and subacute to chronic lesions to days 29–63 following experimental infection [9]. However, different lesion stages can be simultaneously observed in one individual.

**Figure 2:**
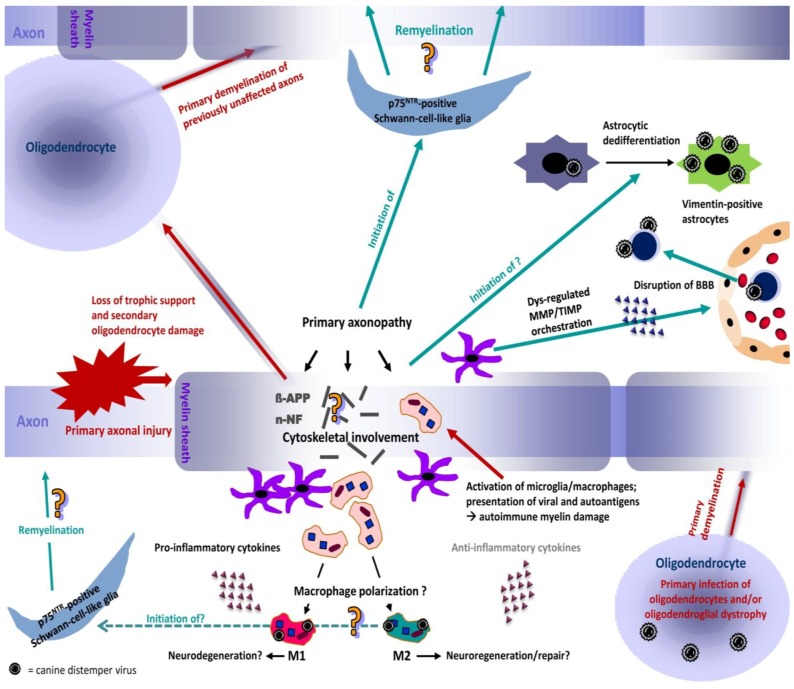
Proposed neuropathogenesis of CDV-DL. The model is based on the concept of primary axonal injury (inside-out model), leading to disturbances in the axonal cytoskeleton and axonal transport mechanisms. The axonal damage induces secondary myelin loss and may induce oligodendrocyte death due to loss of trophic support. In consequence, previously non-affected axons undergo primary demyelination as one oligodendrocyte myelinates multiple axons. Another possible but less frequent mechanism of primary demyelination is the primary viral infection of oligodendrocytes. CDV-DL infection does not necessarily lead to oligodendroglial death but may induce an oligodendrocytic dystrophy with reduced translation of myelin proteins leading to demyelination. Axonal and myelin injury leads to activation of microglia/macrophages, phagocytosis of myelin fragments and presentation of viral antigens and autoantigens, potentially triggering another cascade of autoimmune mediated bystander demyelination presumably by “epitope spreading”. Resident activated astrocytes and microglia as well as attracted leukocytes from the peripheral blood can produce pro- and anti-inflammatory cytokines that can favour either neurodegeneration or neuroregeneration, respectively. The role of potential macrophage polarization towards a neurodegenerative (M1) or -regenerative (M2) phenotype remains undetermined so far and could represent an important pathogenetic mechanism. In parallel, the evident dysregulation of matrix metalloproteinases (MMPs) and their inhibitors (TIMPs) induces a disruption of the blood-brain-barrier (BBB), which favors the invasion of leukocytes and virus particles via cell associated viremia, which represents one possible mode of neuroinvasion. Dedifferentiation of astrocytes into immature vimentin expressing cells enhances their viral infection, leading to further virus spread. Pre-myelinating p75^NTR^-positive Schwann-cell-like glia arise during CDV-DL, potentially triggered by early axonal damage and activated microglia/macrophages. These cells may represent an important therapeutic target. However, whether manifest Schwann cell mediated remyelination occurs during CDV-DL remains elusive so far.

During the acute stage of CDV-DL, CDV mainly replicates in glial cells of the white matter [42,43]. 95% of all infected cells within acute CDV-DL lesions are astrocytes, highlighting this cell type as the major target during the early stages of CDV-DL [22]. Besides astrocytes, viral antigen is additionally evident in neurons, microglial cells as well as ependymal, leptomeningeal, and plexus chorioideus cells [4,44]. Interestingly, oligodendrocytes neither show considerable necrotic nor apoptotic changes following CDV infection and only very few of them contain CDV antigen [45,46,47]. However, a low proportion of oligodendrocytes (8%) contain CDV nucleic acids [45]. Thus, this restricted oligodendroglial infection without evidence of viral protein production may result in metabolic changes, subsequently leading to demyelination [45,46] (Figure 2). Whether this potential oligodendrocyte dystrophy is directly virus-mediated remains to be proven. Evidence for oligodendroglial dystrophy during CDV-DL has recently additionally been shown on the transcriptome level, where multiple myelin genes exhibited a selective down-regulation during subacute CDV-DL [9].

Interestingly, the amount of detectable virus antigen decreases with the duration of disease, accompanying the increasing inflammation in an antiparallel manner [44]. Especially the diminished expression of envelope proteins might indicate restricted viral infection of brain cells in the advanced disease stages. This is believed to be a contributing factor to persistence of CDV, which is mainly evident in white matter areas outside the inflammatory foci [48].

Immunopathologically, antibody-independent cytotoxicity may be involved in the pathogenesis of acute stages of CDV-DL, whereas delayed type immune-mediated processes with antibody-dependent T cell cytotoxicity are suggested to play a role in chronic stages of CDV-DL [39,49,50]. Demyelination in advanced disease phases is believed to be no longer directly virus-induced, but rather represents an immunopathological bystander phenomenon in a biphasic disease process [9,51]. Numerous studies have consequently focused on the immunopathological basis of CDV-DL in order to elucidate the molecular mechanisms that contribute to lesion initiation and progression, respectively. Here, a derailment of the expression of cytokines and MMPs seems to play a fundamental role in the fate of CDV-DL lesions.

### 3.2. Dominance of Pro-Inflammatory Cytokines in CDV-DL — A Hint for Macrophage Polarization?

Marked alterations in the molecular microenvironment accompany the temporal disease course of CDV-DL. The detailed role of cytokines in CDV-DL has been reviewed recently [51]. Briefly, an impressive up-regulation of several pro-inflammatory cytokines has been demonstrated in brain tissue of dogs suffering from CDV-DL, and a similar dominance of certain pro-inflammatory cytokines can be appreciated in the CSF and blood. In the spleen of dogs suffering from CDV-DL, increased mRNA-expression of tumor necrosis factor (TNF) is observed in parallel with depletion of Foxp3+ regulatory T cells, while transcription of interleukin (IL)-2 is decreased in the acute disease phase [52].

In the brain, some prototype pro-inflammatory mediators such as interleukin (IL)-8, IL-6, IL-1, and tumor necrosis factor (TNF) are expressed in the very early disease phase prior to leukocyte infiltration, and, thus, resident CNS cells such as microglia and astrocytes are suggested to be the primary source [51]. Moreover, early cytokine expression appears to be directly virus-mediated. Indeed, cytokine induction correlates with the degree of virus production of canine CNS cells *in vitro* [53]. The striking dominance of pro-inflammatory cytokines with lacking expression of anti-inflammatory cytokines might favor disease progression and the progressive development of demyelination in advanced disease phases. The pro-inflammatory shift of cytokines in CDV-DL is highly similar to canine spinal cord injury (SCI), which is also characterized by a dominating expression of pro-inflammatory cytokines [54]. Similar to experimental SCI in rodents [55], experimental models of demyelinating diseases such as acute experimental autoimmune encephalomyelitis (EAE) are accompanied by a polarization of microglia/macrophages into a neurotoxic classically activated M1 phenotype, whereas the proportion of alternatively activated M2 macrophages, potentially associated with disease remission, is comparatively lower [56]. While classically activated M1 macrophages are characterized by secretion of pro-inflammatory cytokines, such as TNF, IL-1, IL-6, and IL-12, alternatively activated M2 microglia/macrophages possess an anti-inflammatory cytokine signature, including the expression of TGF-β and IL-10 [57]. In fact, a shift towards the pro-inflammatory M1 phenotype promotes relapsing EAE, while administration of alternatively activated M2 monocytes has proven to suppress severe EAE [58], indicating that modulation of the M1/M2 ratio may represent a promising therapeutic goal in certain demyelinating diseases. Microglia/macrophages also play a pivotal role in CDV-DL, especially in advanced disease phases. Major histocompatibility complex class II up-regulation is predominantly seen on microglia in chronic CDV-DL [49]. Moreover, microglia/macrophages are immunoreactive with antibodies against IL-1 in inflammatory CDV-DL lesions [59]. The dominance of pro-inflammatory cytokines in CDV-DL could thus similarly indicate a polarization of macrophages, possibly into the M1 phenotype, during CDV-DL (Figure 2). However, phenotype polarization of canine microglia/macrophages has not been investigated yet, and may represent an interesting approach for future studies.

In addition to dominating expression of pro-inflammatory cytokines, additional events such as disruption of the BBB may represent necessary factors to cause obvious histopathological changes during CDV-DL [60,61]. In this respect, a number of recent studies have highlighted MMPs and their inhibitors as additional crucial proteins in the molecular pathogenesis of nervous distemper [61,62,63].

### 3.3. The Role of Matrix Metalloproteinases and Their Inhibitors in CDV-DL

As mentioned before, CDV and inflammatory cells can enter the brain hematogenously by crossing the BBB (Figure 2) [64]. This process is in part facilitated by proteolytic enzymes, namely MMPs [65]. MMPs are zinc-dependent endopeptidases, which are important regulators of extracellular matrix metabolism and additionally function as activators of several cytokines and chemokines [66,67,68]. They are controlled by tissue inhibitors of matrix metalloproteinases (TIMPs) and other proteins such as the reversion-inducing cysteine-rich protein with Kazal-motifs (RECK) [69,70].

Spontaneous CDV infection leads to changes in the expression of certain MMPs and TIMPs, depending on the stage of the disease [61,71,72]. Most notably, and similar to observations on cytokine expression, a prominent up-regulation of several MMPs and TIMPs is observed in acute and subacute lesions prior to inflammatory changes. Immunohistochemical double-labeling techniques reveal that MMPs and TIMPs are mainly expressed by microglia/macrophages and astrocytes [72]. Substantiating these cells as an important source, microglia isolated from dogs with inflammatory CNS diseases, including CDV-DL, contain a higher number of MMP-9 mRNA transcripts compared to microglia from dogs suffering from non-inflammatory CNS diseases. This might account for a facilitated invasion of white blood cells into the CNS [63]. However, a canine macrophage/monocyte cell line (DH82 cells) persistently infected with CDV, displays only minimal variations in the number of MMP-9 mRNA transcripts compared to non-infected DH82 cells [62]. Although glial cells seem to play an important role in the regulation of the extracellular matrix integrity, few studies focused on the MMP and TIMP expression of different glial cell types *in vitro*. Non-infected primary canine olfactory ensheathing cells (OECs), peripheral Schwann cells, and p75 neurotrophin receptor (p75^NTR^)-positive glial cells display a higher number of MMP-2, MMP-9, and TIMP-1 mRNA transcripts than fibroblasts (data not shown). Nevertheless, there are some variations between the different glial cell populations with respect to detectable transcripts. Following CDV infection, few, inconsistent changes in the gene expression pattern are observed in primary canine glial cell cultures (data not shown).

With lesion progression a decrease in the expression of the vast majority of MMPs and TIMPs is noted, whereas MMP-11, -12, and -13 exhibit constant expression levels [72]. In subacute and chronic CDV-DL plaques with inflammation, perivascular mononuclear cells express MMPs and TIMPs and thus function as another contributing source in addition to astrocytes and microglia [72]. Similarly, disease stage dependent changes in the MMP and TIMP expression are observed on the transcriptional level as demonstrated by *in situ* hybridization and RT-qPCR [61]. While control dogs display a comparatively weak signal for MMP-9, -14, and TIMP-1 in neurons, astrocytes, microglia, and oligodendrocytes, a high number of strongly mRNA-expressing cells are found mainly in subacute and chronic inflammatory lesions of dogs suffering from CDV-DL [61]. Compared to the increase in MMP-9 and MMP-14, a lower increase in the number of TIMP-1 mRNA expressing cells is seen [61]. Furthermore, high levels of MMP-9 and -12 mRNA transcripts in non-inflammatory CDV-DL lesions are strongly associated with abundant levels of CDV antigen (data not shown), which might indicate an initial, directly virus-mediated degradation of the extracellular matrix contributing to demyelination. In subacute, non-inflammatory lesions a high number of RECK mRNA transcripts is observed in addition to TIMP-1 mRNA transcripts (data not shown). In combination with the prominent astrogliosis at this disease stage, this possibly leads to an inhibition of matrix degeneration in the close proximity of these glial cells. Consequently, TIMP-1 and RECK might represent enzymes that prevent lesion progression and myelin loss. In addition to the cerebellar parenchyma, where elevated amounts of pro-MMP-2 and pro-MMP-9 are noted, high levels of pro-MMP-2 are also found in the CSF of dogs with subacute CDV-DL [73]. Interestingly, active MMP-2, required for cellular migration across the BBB, is present in the CSF of some naturally CDV infected dogs [73]. Similar findings are reported for canine steroid-responsive meningitis-arteritis (SRMA), where a transcriptional up-regulation of MMP-2 in CSF mononuclear cells compared to the expression in blood mononuclear cells suggests a relevant influence of MMP-2 for inflammatory cell migration through the blood-CSF-barrier [74]. Furthermore, the hyaluronate receptor CD44, a cell surface receptor not only for hyaluronate but also for MMPs, is up-regulated in a plaque-associated manner in acute and subacute CDV-DL lesions [75,76]. In chronic lesions, CD44 expression decreases in parallel to a reduction in glial fibrillary acidic protein (GFAP)-positive astrocytes but retains expression in perivascular mononuclear cells [75]. In summary, the retained CD44 expression in perivascular mononuclear cells in chronic CDV-DL lesions might contribute to the increased expression of MMPs and their inhibitors in chronic lesions and may thus display a marker for immune cell activation. Taken together, these studies indicated an imbalance in the MMP and TIMP expression as an additional crucial factor for lesion initiation and progression during CDV-DL pathogenesis especially in terms of hematogenous and subsequent astrocytic infection [61,72,76]. In fact, astrocytes have proven to be a key cell type involved in the pathogenesis of both the early and late stages of CDV-DL. In detail, plasticity changes in their maturation state, which will be detailed in the following paragraph, may represent an additional important factor in CDV-DL pathogenesis.

### 3.4. The Role of Astrocytic De-Differentiation in Chronic Lesions — Another Underestimated Mode of Virus Persistence and Spread?

Once CDV has entered the brain, astrocytes represent one of the major targets for infection and play an important role in neuroinvasion and cell-to-cell spread via astrocytic processes [22,77]. It is well known that white matter demyelination is followed by astrocytic hypertrophy [78], and occasionally formation of astrocytic syncytia, reactive gemistocytes and isomorphic gliosis may be observed [39,79]. In acute CDV-DL lesions, viral replication predominantly occurs in astrocytes [43,80]. Based on the developmental stage, astrocytes express three types of intermediate filaments such as GFAP, vimentin, and nestin. Nestin and vimentin are the main intermediate filament proteins in immature astroglial cells, whereas maturing astrocytes embody vimentin and adult astrocytes contain only GFAP. Around birth, a switch from vimentin to GFAP expression takes place. Thereby, vimentin is progressively replaced by GFAP in differentiated astroglial cells that transiently co-express both proteins [81,82]. Vimentin seems to precede the expression of tissue-specific GFAP in the developing human spinal cord and a co-expression of both proteins continues to the mature state of this cell lineage [83]. In addition, GFAP and vimentin expression is observed in activated astrocytes of reactive gliosis in trauma, tumor, or neurodegenerative disorders affecting the CNS [84,85,86,87]. In fact, the hallmark of reactive gliosis in CNS ischemia, trauma or neurodegeneration is a characteristic hypertrophy of cellular processes of astrocytes and upregulation of GFAP and vimentin and a reexpression of nestin, all participating in the formation of the intermediate filament network [88].

Furthermore, GFAP and vimentin expression are required for the proper formation of a glial scar [87,89,90]. These glial scars constitute both a mechanical and a chemical barrier that block nerve regeneration and axonal growth [91]. In addition, vimentin may stabilize the formation of GFAP intermediate filaments in reactive astrocytes [92] and may act as a cytoskeleton-associated protein [93].

The antigenic profile of so called “scar astrocytes” of chronic MS lesions consists of various proteins including GFAP and vimentin, which is interpreted as a significant modification of astrocytes protein expression in those lesions [94]. In Alzheimer`s disease, Pick’s disease, amyotrophic lateral sclerosis, MS, and cerebral infarction numerous intensively vimentin-immunopositive astrocytes displaying both protoplasmic and fibrous morphology are reported [36,95]. In dogs, other inflammatory CNS lesions such as necrotizing leukoencephalitis, also show astroglia-like cells expressing vimentin within the malacic lesions. Furthermore, a part of this cell population displays a gemistocytic phenotype [96]. In a case of old dog encephalitis, vimentin-positive astrocytes have been detected in areas with rarefication, as well as in adjacent tissue [36].

**Figure 3:**
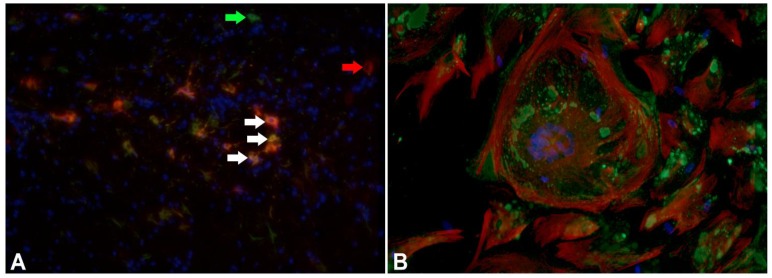
Immature astrocytes are characterized by expression of vimentin in CDV-DL. (**A**) Detection of glial fibrillary acidic protein (GFAP; green; Cy2-labeled secondary antibody; green arrows) and vimentin (red; Cy3-labeled secondary antibody; red arrow) expression in a chronic distemper lesion. Co-localization of GFAP and vimentin is displayed by a yellowish color in the cytoplasm of astrocytes (white arrows). Counterstaining of nuclei with bisbenzimide (blue). 200 fold magnification; (**B**) Immunostaining for vimentin and CDV antigen in adult canine mixed primary cell cultures infected with CDV strain R252 at 30 days post infection. Vimentin expression in mono- and multinucleated cells characterized by a fine fibrillary network (red: vimentin, Cy3-labeled secondary antibody). CDV antigen is mainly found in the cytoplasm (green: CDV nucleoprotein, Cy2-labeled secondary antibody). 400 fold magnification.

Following these observations, the expression of GFAP and vimentin was investigated in acute, subacute, and chronic lesions of CDV-DL [97]. A distinct co-expression of GFAP, the major intermediate filament of mature astrocytes, and vimentin, one of the main intermediate filaments in immature astrocytes, in subacute and chronic lesions was noted [97]. Surprisingly, CDV antigen was detected in GFAP expressing astrocytes in early but not in advanced lesions. In subacute and chronic lesions, CDV antigen was found in vimentin expressing cells with the morphological appearance of astrocytes (Figure 3) [97]. Similar findings could be recapitulated *in vitro*, where canine cell cultures were composed of GFAP-positive astrocytes, vimentin-positive cells and other glial cells (Figure 3A). Following infection with the CDV-R252 strain, GFAP-positive astrocytes displayed considerable cytopathic effects such as formation of multinucleated syncytial giant cells, which were characterized by a disrupted cytoskeleton with condensation and spoke wheel-like structure formation of filaments, whereas vimentin-positive cells though more frequently infected did not show any alteration in the filament network [97]. This indicates increased vulnerability of mature GFAP-positive astrocytes compared to vimentin-positive astrocytes in terms of increased intermediate filament disruption in mature astrocytes [97]. Vimentin may in fact influence the formation of other intermediate filaments with either nestin or GFAP as obligatory partners in the normal adult mouse brain either by co-polymerization with other intermediate filaments or in an indirect way [98]. Using primary astrocyte cultures of vimentin knock-out mice, it was pointed out that vimentin appeared to be necessary to stabilize GFAP filaments and the intermediate filament network formation [92]. Accordingly, *in vitro* transfection experiments showed that the growth of astrocytes is inhibited when vimentin expression is decreased [99].

The abundant numbers of CDV- and vimentin-positive astrocyte-like cells might thus indicate a change of cell tropism of CDV. Vimentin-positive cells of glial or nonglial origin should, thus, be considered as a contributing factor in advanced distemper lesions and might represent a new target for virus infection (Figure 2). In addition, virus infection of vimentin-positive cells could be related to the occurrence of a new, histogenetically different cell type or may be related to defects in virus replication, transcription and translation due to intrinsic and extrinsic factors. Moreover, it can be assumed that due to maintenance of the BBB, clearance of extracellular ions and neurotransmitters and direct metabolic support for oligodendrocytes, astrocytes contribute directly or indirectly to demyelination [100], though the exact role of astrocytes in the pathogenesis of demyelination in CDV-DL remains to be determined.

### 3.5. Early Axonal Damage as a Pivotal Triggering Mechanism — The End of an Old Dogma?

**Figure 4:**
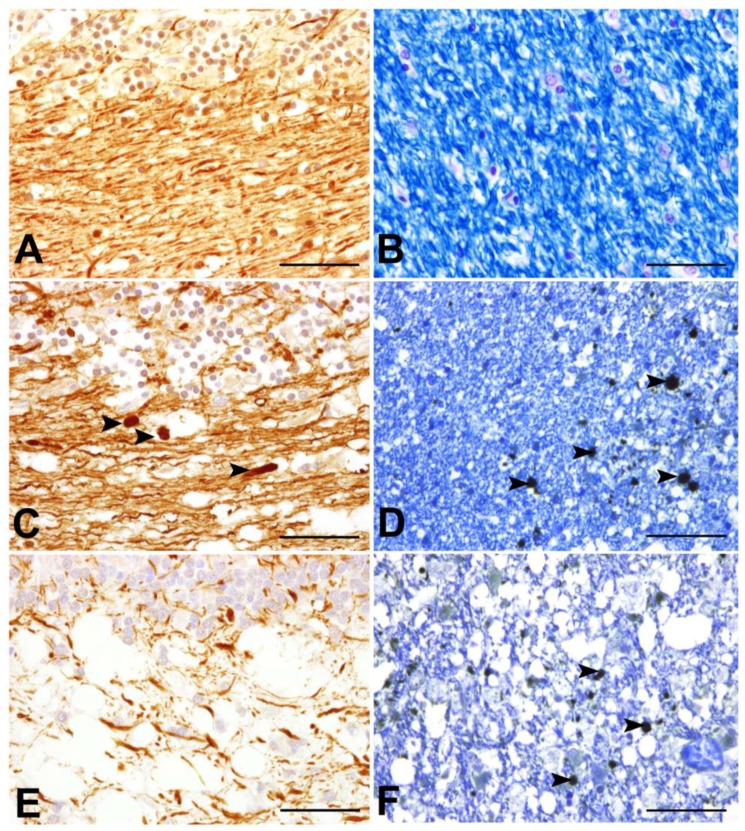
Axonal pathology during the time course of CDV-DL. (**A**), (**B**) cerebellum of control dogs; (**C**),(**D**): acute lesions of canine distemper virus induced demyelinating leukoencephalitis (CDV-DL); (**E**),(**F**) subacute lesions of CDV-DL; (**A**),(**C**),(**E**) Immunohistochemical labelling of phosphorylated neurofilament (p-NF) in the cerebellar white matter; (**B**),(**D**),(**F**) Luxol Fast Blue staining for myelin in the cerebellar white matter; (**D**),(**F**) Double-labelling of myelin with LFB and immunohistochemical detection of beta amyloid precursor protein (β-APP). (**A**) Expression of p-NF in the cerebellar white matter of a control dog. Note the normal density of axons, which physiologically express p-NF; (**B**) LFB staining reveals normal myelin content in the cerebellar white matter of a control dog; (**C**) Expression of p-NF in the cerebellar white matter in an acute lesion of CDV-DL. The morphologically unremarkable axons stain positive for p-NF. Additionally, a slight decrease of the axonal density, represented by loss of p-NF-immunoreactivity is visible. Moreover, single swollen axonal structures also stain positive for p-NF (arrowheads), which might be attributed to accumulation of neurofilaments due to impaired axonal transport; (**D**) In an acute lesion of CDV-DL, staining with LFB demonstrates normal myelin density, while axonally transported β-APP is expressed by several swollen axons (arrowheads) in the cerebellar white matter. This observation is consistent with the inside-out theory based on the assumption of primary axonopathy; (**E**) Expression of p-NF in a subacute lesion of CDV-DL. A marked loss of p-NF-positive axonal structures (decreased axonal density) is evident; (**F**) Expression of β-APP in a subacute lesion of CDV-DL. As an expression of ongoing axonal damage, β-APP-immunoreactive axons (arrowheads) are present within demyelinated areas as demonstrated by reduced staining intensity for LFB. Bar = 50 µm. (**A**),(**C**),(**D**),(**E**),(**F**): Avidin-Biotin-Peroxidase Complex Method, 3.3’diaminobenzidine-tetrahydrochloride.

Morphologically, damaged axons are characterized by swollen structures in dilated myelin sheaths [101,102,103], which contain densely packed axoplasmic organelles (“dense bodies”). In the CNS, axonal lesions share characteristics of Wallerian degeneration, where the distal segment degenerates following focal axonal injury. Subsequently, the proximal segment may undergo retrograde degeneration up to the perikaryon (“dying back”) [103]. Common immunohistochemical markers for axonal pathology consist of antibodies directed against phosphorylated and non-phosphorylated neurofilaments (p-NF and n-NF; Figure 4) and β-amyloid precursor protein (β-APP; Figure 4). Unaltered axons predominantly express p-NF, and immunoreactivity for p-NF has commonly been used as a marker for overall axonal density [10,104]. Following pathological injuries, phosphorylation disturbances lead to increased axonal expression of n-NF [104,105,106,107]. Similarly, β-APP is a highly sensitive marker for early axonal damage [103,108,109,110,111]. This protein accumulates in axons that have undergone functional changes in terms of turbulences in fast axonal transport mechanisms [103,109]. Both β-APP and n-NF expression have previously been observed in damaged axons in acute and chronic MS lesions, in several experimental animal models for MS, as well as in traumatic brain injury and degenerative diseases such as Alzheimer’s disease and amyotrophic lateral sclerosis [110,112,113,114,115,116]. Moreover, accumulation of both proteins has recently been reported in dogs suffering from SCI [117].

The role of axonal damage in CDV-DL has remained enigmatic so far, but recently, axonal damage was demonstrated to occur in both early, yet still myelinated lesions, and chronic demyelinated plaques in dogs suffering from CDV-DL [40]. In addition, a progressively decreasing axonal density during the disease was noticed, as demonstrated by diminished p-NF immunoreactive area [40]. Moreover, β-APP-positive axons were also found in areas expressing CDV antigen, and still lacking visible lesions. However, the amount of β-APP-positive axons reached its maximum in subacute lesions with myelin loss by light microscopy [40].

Interestingly, the recent findings in CDV-DL are highly similar to observations in MS and its animal models. Reduced axonal density is a well-known phenomenon in many experimental models for MS such as EAE in rodents and non-human primates or Theiler’s murine encephalomyelitis virus induced demyelinating disease (TMEV-IDD) in susceptible mice strains [104,106,118,119,120]. Similarly, a selective reduction of axonal p-NF is observed in white matter lesions in the spinal cord of MS patients [10]. In CDV-DL, β-APP pointed out to be a valuable marker for early axonopathy. Comparably, β-APP-positive axons can be detected in acute and to a lesser extent at the periphery of chronic MS lesions [10,110,111].

Conclusively, the recent observations emphasize that axonal damage is an early phenomenon in CDV-DL, which precedes obvious demyelination. This finding has important consequences as it is suggestive of an inside, *i.e*., axon, to outside, *i.e*., myelin sheath, lesion development (Figure 2) [121]. This is in clear contrast to the traditional view of CDV-DL as a purely primary demyelinating disease. In fact, the observations in CDV-DL rather suggest substantial, potentially directly virus mediated primary axonal damage with secondary demyelination, at least in the early disease phases (Figure 2). Additionally, in advanced CDV-DL lesions, primary demyelination of initially preserved axons may be caused by a “bystander” mechanism or by immunopathological processes as an outside to inside lesion (axonal damage follows myelin loss). Moreover, oligodendrocytic death or oligodendroglial metabolic disturbances might lead to primary demyelination of previously unaffected axons [121].

The presented data may indicate that axonal damage represents a key event in the initial disease phase and during the progression of CDV-DL. Further investigations have to focus on the detailed molecular pathogenesis of axonal injury. Another interesting question that remains to be solved is whether there are spontaneous regenerative attempts in CDV-DL. In fact, recently, relatively high ratios of growth associated protein (GAP)-43 and β-APP-positive axonal spheroids were reported in MS, suggestive of a robust intrinsic regenerative response of axons [116]. Moreover, the GAP-43/β-APP ratio correlated with macrophage infiltration, indicating that macrophages might be involved in neuroplastic events during MS [116]. The role of potential spontaneous regenerative mechanisms of axons in CDV-DL is not known so far and should be addressed in future studies. Indeed, novel findings point out that regeneration attempts in terms of potential remyelination efforts may in fact be triggered by early axonal damage during CDV-DL. Recently, a unique type of macroglia has been observed in CDV-DL, which might represent a first hint for endogenous remyelinating events during CDV-DL.

### 3.6. Aldynoglial p75^NTR^-Positive Glial Cells Emerge in Response to CDV Mediated CNS Damage — A First Step in Schwann Cell-Mediated Remyelination?

Failure of sufficient remyelination is the hallmark of demyelinating diseases, though especially in toxic experimental models such as cuprizone-, lysolecithine-, and ethidiumbromide-induced demyelination, remyelination progresses towards nearly complete restoration [122]. Failure of remyelination seems to be rather attributed to the nature of a specific disease entity than to a general incompetence of the organism to remyelinate demyelinated areas [123]. In fact, remyelination is a common phenomenon in MS, even if it does not lead to complete recovery [123]. In this respect, so called shadow plaques are observed in early MS lesions. These areas of complete remyelination are characterized by paler myelin staining, which results from a general reduction of myelin sheath thickness throughout the lesion [124]. Remyelination during CDV-DL has not been a topic of extensive research so far and actually lacks confirmation [3].

The generation of mature oligodendrocytes is mediated by central precursor cells termed oligodendrocyte precursor cells (OPCs), which differentiate into myelinating oligodendrocytes [123,125,126]. OPCs are characterized by the expression of stem cell markers such as the proteoglycan NG2, Olig2, and platelet derived growth factor receptor α (PDGFR-α) [127,128,129]. Although these precursors are abundant, even in the adult CNS, naturally occurring demyelinated lesions are believed to contain factors that inhibit precursor differentiation into remyelinating oligodendrocytes [122].

**Figure 5:**
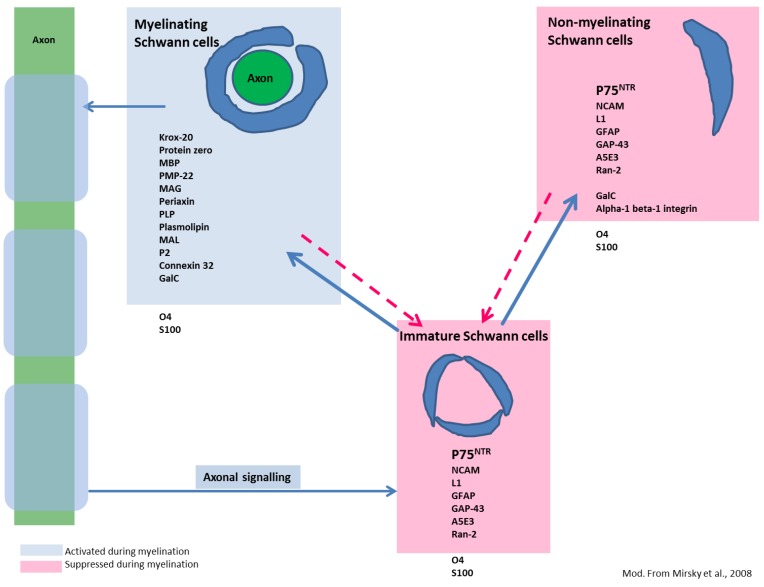
Differentiation stages of Schwann cells and commonly expressed molecules. Unlike oligodendrocytes, immature Schwann cells may differentiate into two distinct phenotypes, myelinating (left) and non-myelinating (right) Schwann cells. However, differentiation is reversible as indicated by the dotted arrows. Blue boxes represent markers of myelinating Schwann cells and molecules promoting their differentiation. All of these markers display strong up-regulation in the stage of myelination. Red boxes include markers of non-myelinating and immature Schwann cells, which are down-regulated during myelination. Note that p75^NTR^ is expressed in all differentiation stages except in myelinating Schwann cells. Whether the detected p75^NTR^ expressing bipolar cells in CDV-DL differentiate into myelinating Schwann cells is currently not known. Markers and scheme modified from Mirsky *et al*., 2008 [141].

It is a well-known phenomenon that, under certain circumstances, demyelinated CNS axons are remyelinated by Schwann cells [130]. Peripheral type myelination of demyelinated CNS axons by Schwann cells has been reported in traumatic human SCI, as well as in MS and its animal models, such as toxin-induced demyelination and TMEV-IDD [119,131,132,133,134,135]. Interestingly, it is predominantly observed in regions devoid of or at least poor in GFAP expression [133,136,137]. The functional consequence of Schwann cell-mediated remyelination in certain CNS lesions remains enigmatic so far [123], however, recent reports on a comparatively high percentage of Schwann cell-mediated remyelination in comparison to remyelinating events done by oligodendrocytes in a mouse model for SCI [127] highlight CNS Schwann cells as a potentially clinically relevant target. Interestingly, recent observations in mice suggest that Schwann cells and oligodendrocytes remyelinate separate axonal populations with large caliber axons being predominantly remyelinated by Schwann cells, including 50% of the axons exhibiting a diameter of more than 2 µm [127]. It is concluded that Schwann cells may in fact be essential for quick and early neuroprotection following CNS demyelination, especially for large diameter axons [127]. In the peripheral nervous system, all differentiation stages of Schwann cells express p75^NTR^ with the exception of myelinating Schwann cells (Figure 5) [138,139,140,141]. Unlike oligodendrocytes, two distinct types of Schwann cells are known, non-myelinating and myelinating Schwann cells [138]. During myelination, Schwann cells down-regulate the expression of p75^NTR^ and up-regulate peripheral myelin-specific molecules such as periaxin and myelin protein zero (P_0_) (Figure 5) [127,129,138,142]. Even though p75^NTR^ is not expressed by the myelinating Schwann cell stage, there is vast consensus that p75^NTR^ signaling plays a crucial role in the mediation of myelination through Schwann cells [138,139].

Whether effective Schwann cell remyelination occurs in CDV-DL is unknown so far. However, a glial cell type with Schwann cell-like properties in adult canine brain was identified *in vitro* as early as 2008, where these cells displayed a spindle-shaped morphology three weeks after seeding [143]. Strikingly, the role of p75^NTR^ expression has recently been described in dogs suffering from naturally occurring CDV-DL, highlighting this molecule as a potential candidate for endogenous regenerative events following CDV infection [41]. While in non-infected control dogs p75^NTR^ expression is limited to blood vessels and to the periphery of leptomeningeal vessels, p75^NTR^ is additionally expressed on bipolar, spindle-shaped cells, morphologically reminiscent of Schwann-like cells in dogs with CDV-DL [41]. In addition and not observed in control dogs, a comparatively low expression is noted on stellate-shaped cells, suggestive of microglia. The demonstration of a lacking co-localization with markers specific for astrocytes, microglia, and mature oligodendrocytes substantiates that the detected p75^NTR^-immunoreactive bi- to multipolar cells in CDV-DL lesions represent a distinct and unique glial population, similar to p75^NTR^ expressing macroglia *in vitro* [143]. Despite observations *in vitro*, which demonstrate high susceptibility of p75^NTR^ positive macroglia for CDV infection, there is no evidence that p75^NTR^-positive cells are infected by CDV *in situ* as demonstrated by lacking immunohistochemical co-localization of CDV antigen and p75^NTR^ [41,143]. The highest number of p75^NTR^-positive glia is seen in dogs with subacute lesions with remarkable inflammation. However, though relatively lower in number, similar cells are already observed in early CDV-DL lesions prior to the onset of demyelination. This interesting finding implicates that factors other than demyelination itself might function as triggering events that contribute to the differentiation of such cells. Interestingly, p75^NTR^ expression on Schwann like glia strictly parallels axonal pathology, as demonstrated by immunoreactivity of axons for n-NF and axonal expression of β-APP [41]. Thus, early axonal damage might represent a pivotal initial pathogenetic mechanism that triggers the occurrence of these cells (Figure 2) [41].

Another interesting question, which remains to be solved, is related to the origin of the detected cells. If the detected cells in CDV-DL in fact represent Schwann cells, where do they derive from? It has long been assumed that Schwann cells detected in other demyelinating diseases, derive from Schwann cells associated with perivascular nerves, meningeal nerves or the spinal roots [130,144,145]. However, recent studies using Cre-lox fate mapping in transgenic mice in a lysolecithin-induced demyelination model demonstrated that PDGFR-α/NG2 expressing CNS progenitor cells, thus suggestive of OPCs, give rise to both oligodendrocytes and Schwann cells [129]. In fact, the majority of Schwann cells observed in remyelinated areas were proven to originate from these CNS precursors and not from invading cells from the periphery [129]. These recent observations highlight OPCs as an interesting target, which shares characteristics of adult stem cells. In fact, OPCs have shown to be capable of giving rise to oligodendrocytes, astrocytes, neurons, and lastly, centrally derived Schwann cells [123]. More recently, a transcriptome analysis revealed a strong similarity of cultivated canine Schwann cell like brain glia with both OECs and peripheral Schwann cells *in vitro* [146]. Promotion of the differentiation of OPCs into one lineage and suppression of other lineages may therefore represent a valuable approach to favor remyelination in demyelinating diseases.

Whether the detected p75^NTR^ positive cells in CDV-DL favor remyelination is currently not known. The results should be interpreted in light of the fact that the possibility of remyelination in CDV-DL is so far solely based on the observation of p75^NTR^ immunoreactivity of a certain cell type, indicating that future studies have to show the true identity and potential of these cells. Both the origin and fate of these cells, especially focused on a potential remyelinating capability, will, thus, represent highly interesting fields for future studies. However, the observation of p75^NTR^-positive glia raises hope for endogenous and spontaneous remyelination in CDV-DL, which might have important therapeutic implications. A major drawback in this conclusion, however, is represented by the fact that none of the p75^NTR^-positive glia detected in CDV-DL were positive for P_0_ and Krox20, both markers for myelinating Schwann cells [41]. Thus, the detected macroglia seem to be arrested in a pre-myelinating stage. Whether they may differentiate into fully competent, myelinating cells and thus significantly contribute to remyelination, potentially in more advanced lesions of CDV-DL, remains to be investigated. Early axonal damage seems to play a significant role as a triggering event, favoring the occurrence of such cells. However, other, to date unknown, factors may play a likewise role in the initiation of Schwann cell occurrence in CDV-DL. In fact, activated microglia/macrophages labeled by BS-1 lectin are consistently seen in areas where p75^NTR^-positive glia appear. This directs to the possibility that macrophages/microglia may play an additional role in the facilitation of p75^NTR^ expressing cells. The use of an interesting *in vitro* model, which shares striking pathological similarities with CDV-DL may contribute to elucidate these potential mechanisms.

## 4. Organotypic Slice Cultures of the Canine CNS — A Promising *in Vitro* Tool for CDV-DL Research?

Organotypic slice cultures of the adult canine CNS have recently been introduced as a promising *in vitro* tool to study the complex interplay of resident glial cells following CNS injury and stress [54,117,147]. Representing an intermediate model between the complex environment *in vivo* and highly simplified dissociative cell cultures, slice cultures are characterized by preserved organotypic morphology with the advantages of *in vitro* models such as easy reproducibility and the avoidance of animal experiments [148,149]. Furthermore, the depletion of peripheral blood supply allows focus on glial cell responses during injury and stress without the complex interference with inflammatory cells [54,150,151].

The production of slices is necessarily accompanied by severe mechanical injury and axonal transection, respectively. Axonal damage has been shown to be characterized by axonal expression of n-NF but not β-APP in cultivated canine spinal cord slices [117]. Moreover, cultivation of adult canine spinal cord and olfactory bulb slices has been linked to pathological alterations such as a remarkable response of phagocytic microglia/macrophages, which is surprisingly similar to original degenerative CNS diseases such as SCI and CDV-DL, respectively [41,54,117]. Following the hypothesis that both axonal damage and microglia/macrophage responses may trigger the occurrence of p75^NTR^-positive glia, the hypothesis arose that similar cells may consequently be observed in cultivated slices of the canine CNS. Interestingly, the occurrence of p75^NTR^-positive bi- to multipolar Schwann like cells following a 10-day cultivation period of canine olfactory bulb slices in parallel with increasing numbers of phagocytic microglia/macrophages was recently reported [41]. Consequently, similar results in cultivated olfactory bulb slices in comparison to natural CDV-DL lesions substantiate axonal pathology as well as the response of microglia/macrophages as important co-factors, potentially favoring the formation of p75^NTR^-positive glia [41]. Similarly, deprivation of axonal contact induces up-regulation of p75^NTR^ on OECs as shown in olfactory bulb slices of dogs in another study, underlining the interdependence of glial p75^NTR^ expression and axons [152].

**Figure 6:**
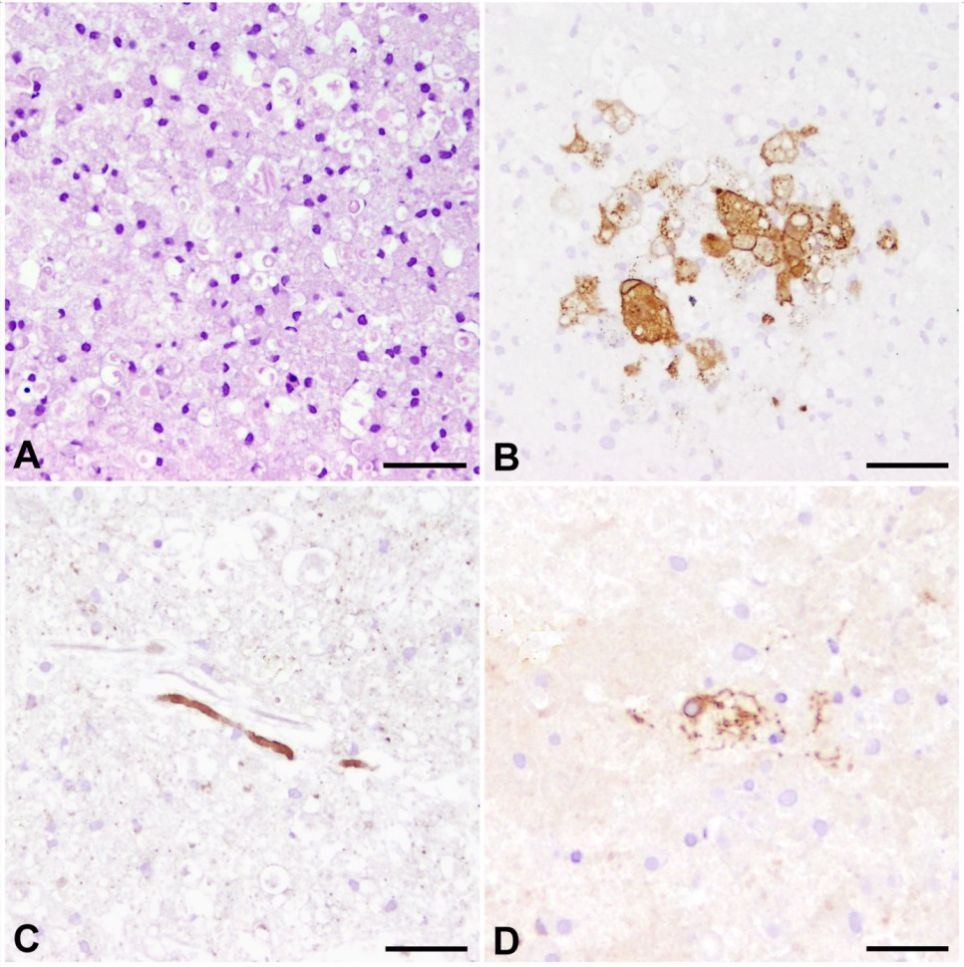
Organotypic slice cultures of the canine cerebellum, infected with the R252 strain of CDV and cultivated for nine days. (**A**) Hematoxylin and eosin staining reveals a remarkable response of activated, large phagocytic Gitter cells, presumably originating from resident microglia; (**B**) Immunohistochemistry for CDV shows that CDV-antigen is predominantly expressed by large, phagocytic cells reminiscent of microglia/macrophages. Whether these cells are infected by replicating virus or if they have phagocytozed viral antigen, remains to be determined in future studies; (**C**) Highly similar to axonopathy in spontaneous CDV-DL, axonal damage is characterized by enhanced expression of non-phosphorylated neurofilament. Similar axonal expression of n-NF is however also observed in non-infected slices, presumably due to mechanical transection of axons as a result of slice preparation; (**D**) p75^NTR^-positive bi- to multipolar cells emerge in response to prolonged culturing, potentially indicating occurrence of pre-myelinating Schwann cells. (**B**–**D**) Avidin-Biotin-Peroxidase Complex Method, 3.3’diaminobenzidine tetrahydrochloride. Bars = 50 µm.

There are currently no published reports on experimental infection of organotypic tissue slices from dogs. In light of the striking similarity of pathological changes in cultivated CNS slices and the *in situ* situation in CDV-DL we sought to determine if organotypic brain slice cultures are capable of being infected with CDV. In fact, we were recently able to demonstrate immunoreactivity for the virulent strain R252 in infected CNS slices of adult dogs cultivated for up to 18 days (Figure 6) (data not shown).

However, the immunohistochemical demonstration of virus antigen is predominantly observed in cells reminiscent of phagocytic microglia/macrophages (Figure 6B). Whether these cells are indeed infected by replicating virus or if the immunopositivity of these cells is based on phagocytozed viral antigens, remains to be determined. Infection of slices is paralleled by the occurrence of p75^NTR^-positive glial cells as well as axonal damage (Figure 6C,D). Similar axonal pathology, characterized by axonal expression of n-NF is however also observed in non-infected slices, as shown in spinal cord slices of healthy adult dogs [117]. Thus, future studies have to clarify if the detected axonopathy in CDV infected slices in fact represents a virus-induced change or is attributed to mechanical axonal transection during slice preparation. However, the overall similarities in terms of axonal pathology, emergence of p75^NTR^ positive glia, and evidence of viral antigen demonstrate that this technique represents a suitable tool for future investigations, as the infection of canine brain slice cultures offers the opportunity for an in-depth insight into the pathogenesis of CDV-DL in the organotypic microenvironment without the need of animal experiments.

## 5. Future Perspectives and Outlook

This review highlighted recent findings and new insights into the neuropathogenesis of CDV-DL with special emphasis of axon-glia interactions and potential modes of remyelination. CDV-DL is accompanied by vigorous changes in the orchestration of cellular signalling mediators such as MMPs and cytokines. The dysregulation of these molecules is an additional pathogenetic factor, which pivotally decides on disease exacerbation and remission, respectively.

Recent investigations demonstrated that axonal and glial changes during the time course of CDV-DL exhibit a strong interdependence. However, it needs to be emphasized that axonal injury represents a crucial and extraordinarily early hallmark of CDV-DL, potentially playing a key role not only in disease progression but also, and maybe more importantly, in disease initiation. The detailed mechanisms of CDV-DL associated axonopathy, however, remain unknown so far. Future studies will help to characterize factors that contribute to axonal damage both on the protein and transcriptomic level. A special focus should consequently be given to cytoskeletal alterations, the role of neurofilament dysorganisation, and axonal transport disturbances in order to understand the molecular basis of axonal damage in CDV-DL in more details. In this context the role of the axonal cytoskeleton in terms of regenerative mechanisms remains to be investigated. Endogenous factors contributing to axonal regeneration as a complex event have been highlighted as an important therapeutic target in various CNS disorders including MS, and might also comprise interesting aspects in the CDV-DL model for demyelinating diseases.

Raising hope for regeneration in CDV-DL, novel observations on p75^NTR^ expression on bi-polar Schwann like cells in CDV-DL highlight the potential of Schwann cell mediated remyelination. Given the fact that remyelination has not been proven in CDV-DL so far, this finding has high relevance for future studies, which, besides the role of p75^NTR^, should concentrate on the expression of peripheral myelin markers such as periaxin and P_0_. Furthermore, the respective roles of oligodendroglial cells and Schwann cells in remyelination and their actual occurrence need to be specified. Similarly, the lineage origin of Schwann cells in the CNS yet needs to be identified. It would be important to know whether they represent resident activated cells originating from OPCs or represent invading cells from the PNS. If future studies indeed succeed in proving substantial regenerative and remyelinating events in CDV-DL, this doubtlessly will have far-ranging therapeutic consequences.

Though the central role of axonal damage as a triggering event is established in CDV-DL, the role of macrophage polarization in CDV-DL is currently unknown. In light of the fact that distinct macrophage responses have been linked to axonal regeneration, degeneration, demyelination, and remyelination, the elucidation of a potential macrophage polarization during CDV-DL appears to represent a promising field for successive investigations.
